# Human Cytomegalovirus Interleukin 10 Homologs: Facing the Immune System

**DOI:** 10.3389/fcimb.2020.00245

**Published:** 2020-06-09

**Authors:** Emma Poole, Tainan Cerqueira Neves, Martha Trindade Oliveira, John Sinclair, Maria Cristina Carlan da Silva

**Affiliations:** ^1^Department of Medicine, University of Cambridge, Cambridge, United Kingdom; ^2^Center for Natural and Humanities Sciences, Federal University of ABC (UFABC), São Bernardo do Campo, Brazil

**Keywords:** human cytomegalovirus, HCMV, HCMV interleukin 10 homolog, UL111A, IL-10 signaling

## Abstract

Human Cytomegalovirus (HCMV) can cause a variety of health disorders that can lead to death in immunocompromised individuals and neonates. The HCMV lifecycle comprises both a lytic (productive) and a latent (non-productive) phase. HCMV lytic infection occurs in a wide range of terminally differentiated cell types. HCMV latency has been less well-studied, but one characterized site of latency is in precursor cells of the myeloid lineage. All known viral genes are expressed during a lytic infection and a subset of these are also transcribed during latency. The UL111A gene which encodes the viral IL-10, a homolog of the human IL-10, is one of these genes. During infection, different transcript isoforms of UL111A are generated by alternative splicing. The most studied of the UL111A isoforms are cmvIL-10 (also termed the “A” transcript) and LAcmvIL-10 (also termed the “B” transcript), the latter being a well-characterized latency associated transcript. Both isoforms can downregulate MHC class II, however they differ in a number of other immunomodulatory properties, such as the ability to bind the IL10 receptor and induce signaling through STAT3. There are also a number of other isoforms which have been identified which are expressed by differential splicing during lytic infection termed C, D, E, F, and G, although these have been less extensively studied. HCMV uses the viral IL-10 proteins to manipulate the immune system during lytic and latent phases of infection. In this review, we will discuss the literature on the viral IL-10 transcripts identified to date, their encoded proteins and the structures of these proteins as well as the functional properties of all the different isoforms of viral IL-10.

## Introduction

Viruses have to face many challenges to become established in the host population. Herpesviruses are extremely successful in overcoming such challenges as evidenced by their ability to establish lifelong infection (Sinclair and Poole, 2014; Collins-McMillen et al., 2018). Cytomegalovirus is the largest of the herpesviruses, belonging to the β-herpesvirinae subfamily and is characterized by slow growth and species specificity (Mocarski et al., 2007).

During primary HCMV infection there is a robust activation of both innate and acquired immune responses which control virus replication in immunocompetent hosts, generally resulting in asymptomatic infection or mild disease. However, infection is not cleared and, as with all herpesviruses, HCMV is maintained for the life time of the host in equilibrium with the host immune system as a latent infection, in certain cell types, with spontaneous subclinical reactivation events which are well-controlled by a normal host immune response (Sinclair and Poole, 2014; Collins-McMillen et al., 2018; Elder and Sinclair, 2019). It is also likely that, *in vivo*, sites of low level persistent lytic infection exist (Goodrum et al., 2012). However, dysregulation of the host immune system can result in clinical reactivation leading to a variety of diseases, highlighting the importance of a competent immune system in the control of the virus (Varani and Landini, 2011; Griffiths et al., 2015). This capacity of HCMV to remain in balance with the immune system is the result of over 200 million years of coevolution with its host where, in order to survive, the virus has acquired a number of molecular mechanisms which allow it to evade anti-viral immune responses (McGeoch et al., 1995; Jackson et al., 2011; Noriega et al., 2012; Wills et al., 2015; Patro, 2019).

During lytic infection all classes of viral genes are expressed, viral DNA is replicated and new infectious viral particles are produced. In contrast, during latency the viral genome is maintained as an episome in the cell nucleus in the absence of viral DNA replication and production of viral particles (Taylor-Wiedeman et al., 1991; Mendelson et al., 1996; Hahn et al., 1998; Reeves and Sinclair, 2013; Elder and Sinclair, 2019). Robust lytic infection can occur in many differentiated cell types, such as smooth muscle cells and fibroblasts, while low level persistence is thought to occur some epithelial and endothelial cells (Sinzger et al., 2008). In contrast, latency is restricted to a few undifferentiated cells. One of the most highly characterized latency and reactivation systems is in the hematopoietic lineage, where latency is maintained in CD34+ progenitors and their derivative CD14+ monocytes. Latency is then broken upon terminal differentiation of these undifferentiated myeloid cells to macrophages or dendritic cells (Taylor-Wiedeman et al., 1991, 1994; Mendelson et al., 1996; Hahn et al., 1998; Slobedman and Mocarski, 1999; Reeves et al., 2005).

During latency, expression of key viral genes required for efficient lytic infection, such as the viral major immediate early (IE) genes, are repressed. The latency-associated transcriptome is currently under intense investigation (Cheng et al., 2017; Shnayder et al., 2018) and single cell RNAseq analyses have recently shown that during latency, far from being silenced, viral gene expression is much more extensive than first thought. A number of latency-associated viral genes, which are all known to also be expressed during lytic infection, have been well-characterized; these include LUNA (latent undefined nuclear antigen; UL81-82as), US28, UL138 (comprising a number of transcripts), and the viral homolog of the interleukin 10 (IL10). RNAs for all these genes have all been identified in natural latency studies and analyses of roles for these genes in experimental latency settings have identified a number of functions for these latency-associated genes (Kondo et al., 1996; Beisser et al., 2001; Goodrum et al., 2002, 2007; Jenkins et al., 2004; Cheung et al., 2006; Hargett and Shenk, 2010; Poole et al., 2013; Humby and O'Connor, 2015; Cheng et al., 2017; Shnayder et al., 2018).

With the expression of viral antigens during both lytic and latent infection, it is clear that the virus must have to continually evade host immune surveillance *in vivo* and, in fact, HCMV is able to perform this task very efficiently through multiple mechanisms (Jackson et al., 2011; Stack et al., 2012). One of the main battles that the virus has to face is to avoid the production of proinflammatory cytokines by immune cells that function to activate the immune system and eliminate the virus (Nordøy et al., 2000; Compton et al., 2003; Clement and Humphreys, 2019). One of the strategies used by HCMV to disable the immune system is to manipulate the immunoregulatory functions of cellular anti-inflammatory interleukin 10 (cIL-10) (Redpath et al., 2001). As part of this strategy, and similar to other herpesviruses, during coevolution with its host, HCMV has ‘captured’ a cIL-10 viral gene (UL111A) which expresses different IL-10 protein isoforms (Kotenko et al., 2000; Lockridge et al., 2000; Jenkins et al., 2004; Lin et al., 2008), which help manipulate the immune response to HCMV.

In this article we review and discuss the transcripts, protein structure and immune subversive mechanisms of the HCMV viral IL10 (vIL-10) isoforms during productive lytic and latent HCMV infections concentrating on its role in modulating infection in the myeloid lineage and comparing it to the structure and functions of human IL10 and other IL-10 homologs encoded by other herperviruses.

## HCMV Infection Upregulates Levels of cIL-10

**Figure 1:**
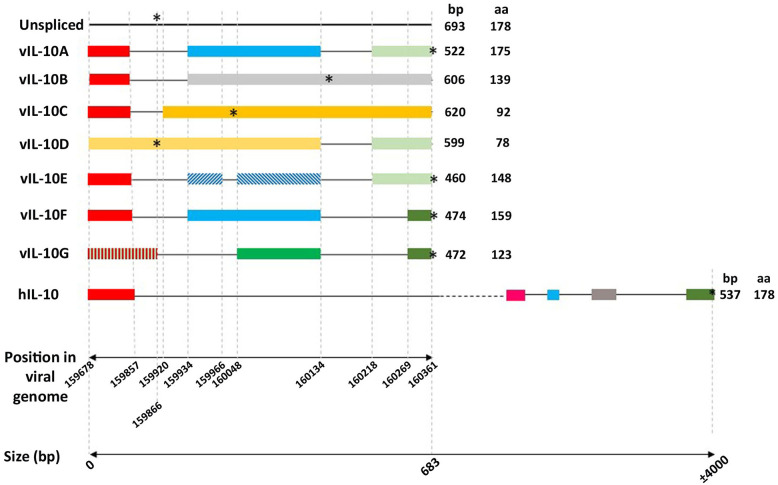
Schematic representation of the genomic intron/exon organization of human IL-10 and the HCMV IL-10s transcripts. Boxes and horizontal lines representing exons and introns were drawn to scale. The 39- and 59-UTRs of cellular IL-10 (GenBank accession no. NP_000563) are not shown. Colored boxes represent homologous exons. The first horizontal scale represents the position in the AD169 HCMV strain genome (GenBank accession number FJ527563.1). The second horizontal scale represents the length in base pairs (bp). Finally, the asterisks represent the position of the stop codons.

cIL-10 is one of the most critical immunoregulatory cytokines of the immune system that acts during inflammatory processes to suppress and control the magnitude of the response in order to avoid excessive immune activation and its consequences (Brooks et al., 2006; Ouyang et al., 2011; Rojas et al., 2017). The human IL10 encoding gene located on chromosome 1 is 5.1 Kb pairs in length and gives rise to a primary transcript containing five exons and four introns. Splicing of this primary transcript generates a 1,629 bp mRNA, including the untranslated regions (UTRs), which produces a protein of 178 aa which is secreted after cleavage of a signal peptide (Vieira et al., 1991; Kim et al., 1992) (Figure 1).

cIL-10 is a homodimer composed of two non-covalently linked monomers that bind to the IL10 receptor (IL-10R) in a coordinated manner. First, the homodimer binds to the high affinity IL-10R1 subunit, changing the conformation of the cytokine, and allowing its binding to the low affinity signaling IL-10R2 subunit (Liu et al., 1994; Kotenko et al., 1997). Binding of cIL-10 to receptor leads to a cascade of intracellular signaling involving the Janus kinases, Jak1 and Tyk2, culminating in activation of the signal transducers and activator of transcription (STATs), which translocate to the nucleus and activate the promoters of various cIL-10 responsive genes (Sabat et al., 2010).

cIL-10 is produced mainly by cells involved in innate and adaptive immunity and acts as a feedback regulator of these cells primarily to suppress the immune response. The main producers of cIL-10 are T cells, in particular the CD4+ T cells, as well as Th1, Th2, and Th17 T cell subsets. cIL-10 is also expressed by T regulatory (Treg) cells, CD8+ T cells and certain subpopulations of B cells (B1 and B2), which are also important players in controlling the inflammatory response. Among the innate immune cells, antigen presenting cells (APCs), including those of the myeloid lineage such as monocytes, activated monocytes, macrophages and dendritic cells (DCs), produce cIL-10 and cIL-10 expression in these cells is regulated by an autocrine feedback to restrict their activation in response to inflammatory cytokines. Natural killer (NK) cells, critical regulators of the innate immune response, are also able to produce cIL-10. cIL-10 has a variety of biological functions in different cell types, but monocytes and macrophages appear to be the main known targets of its anti-inflammatory properties (Sabat et al., 2010; Ouyang et al., 2011). In APCs, cIL-10 is able to inhibit the surface expression of stimulatory and costimulatory molecules as well as the expression of pro-inflammatory cytokines, such as IFNy, TNFα, IL-1b, and IL-6, which can prevent the activation and maintenance of CD4+ T cells. Additionally, cIL-10 has immune stimulatory activities that lead to proliferation of B cells, mast cells and thymocytes (Hedrich and Bream, 2010; Saraiva and O'Garra, 2010; Rojas et al., 2017).

The immunosuppressive functions of cIL-10 are so important that many pathogens, including viruses, regulate the expression of cIL-10 to control the host immune responses in order to overcome antiviral responses and establish latent/persistent infections (Wilson and Brooks, 2011). Perhaps unsurprisingly, HCMV also modulates the infected environment by controlling expression of cIL-10. cIL-10 is upregulated during both latent and lytic phases of HCMV replication, as described below.

It is well-established that increased levels of cIL-10 correlate with viral load during virus reactivation in transplant patients (Nordøy et al., 2000; Cervera et al., 2007; Zedtwitz-Liebenstein et al., 2007; Sadeghi et al., 2008; Essa et al., 2009; Schaffer et al., 2009; Zhang et al., 2009; Krishnan et al., 2010; La Rosa et al., 2011; Limaye et al., 2016). Furthermore, elevated levels of cIL-10 associated with high viral loads, have also been observed in patients with sepsis (Silva et al., 2019) and coronary diseases (Sun et al., 2005). Whilst the specific cell types secreting cIL-10 in response to active viral replication *in vivo* have not been fully elucidated, some studies shown that CD4+ T cells produce cIL-10, in response to lytic and latent antigens *in vivo* (Mason et al., 2012; Schwele et al., 2012; Jackson et al., 2017). It is possible that the induction of cIL-10 acts in favor of the virus to minimize the inflammatory response and, as such, tissue damage caused by virus infection in the immune competent. In an individual infected with HCMV there are continual reactivation events (Reeves and Sinclair, 2008), however, very little tissue damage and this is, perhaps, due to the robust activation of cIL10 by the virus. In support of these observations, studies in mouse and Rhesus models revealed that elevated levels of cIL-10 are important to reduce immune pathologies (Humphreys et al., 2007; Lee et al., 2009; Chang and Barry, 2010; Jones et al., 2010; Mandaric et al., 2012; Clement et al., 2016; Eberhardt et al., 2016).

Given the known immune suppressive functions of cIL-10, it is perhaps not surprising that HCMV infection increases expression of cIL-10 in order to help avoid virus clearance and facilitate persistence. In fact, a recent study by Zischke et al. (2017), provided the first mechanistic evidence of an HCMV protein that can modulate expression of cIL-10 from T cells *in vitro*. It was demonstrated that the extracellular domain of the glycoprotein UL11 binds the CD45 phosphatase (Gabaev et al., 2011), increasing TCR signal strength, and production of cIL-10, via its control over the SRC family kinase LcK (Zischke et al., 2017). UL11 localizes at the surface of infected fibroblasts, therefore the protein likely functions during active replication, *in vivo*, to promote cIL-10 expression (Gabaev et al., 2014). It would be interesting to analyze UL11 variability and test whether there is any correlation with levels of cIL-10 in infected individuals.

Clearly any increases in cIL-10 might be likely to help limit virus clearance, thus favoring a latent infection or low levels of persistence, and therefore the ability of HCMV to induce cIL-10 during active infection would be advantageous to the virus. In fact, induction of cIL-10 occurs during both lytic and latent infection and the viral cIL-10 homologs appear to important modulators of this induction of cIL-10, as further discussed below.

## The UL111A Gene and Different Transcripts

HCMV encodes a cIL-10 homolog gene, which is differentially spliced into several variants. Among them the entitled cmvIL-10 (also termed the “A” transcript) and LAcmvIL-10 (also termed the 'B' transcript) are the most studied. The identification of a cIL-10 gene homolog in the HCMV genome resulted from the work of two groups simultaneously (Kotenko et al., 2000; Lockridge et al., 2000). An ORF in the RhCMV genome with homology to cIL-10 was recognized which was also present in HCMV as well as baboon and African green monkey CMVs (BaCMV, AGMCMV, respectively) (Kotenko et al., 2000; Lockridge et al., 2000). The HCMV transcript, termed cmvIL-10, was shown to contain three exons and two introns (Figure 1), in contrast to four exons and three introns present in the RhCMV transcript. The HCMV cDNA had only 45% sequence identity to the cDNA of RhCMV. The predicted IL10 proteins of HCMV and RhCMV had 27 and 25% identity to their respective host cIL-10 (Kotenko et al., 2000; Lockridge et al., 2000).

A number of cIL-10 homologs have been identified in other members of the herpesviridae family and many of them are located in orthologous locations in the viral genomes, likely indicating a gene capture event in an ancestral virus. Positional orthology is observed in the genus Cytomegalovirus and the cIL-10 capture event is estimated to have taken place at least 42 million ago, when the Old and New World monkeys diverged. Viral cIL-10 homologs have been identified in other members of the herpesviridae family. Many of them are located in orthologous locations within the viral genomes. This likely indicates a gene capture event in an ancestral virus after divergence of subfamilies. The vIL-10s in the genomes of members of the betaherpesvirinae subfamily, including HCMV, rhesus CMV (RhCMV), African green monkey CMV, baboon CMV and cynomolgus CMV, all have high sequence divergence from their host cIL-10 (Marsh et al., 2011; Ouyang et al., 2014). However, gammaherpesviruses, such as Epstein-Barr and Macaque CMV (RhLCV), have 92 and 97% identity to cIL-10 at the amino acid level, respectively (Arrand et al., 1981; Franken et al., 1996; Ouyang et al., 2014). These observations indicate a capture gene event earlier in the coevolutionary history of the betaherpesvirinae subfamily with their hosts (Ouyang et al., 2014).

During lytic HCMV infection in fibroblasts the cmvIL-10 transcript is expressed with late (γ) gene kinetics (Chang et al., 2004) (also more recently classified as TP5 kinetics; Weekes et al., 2014; Nightingale et al., 2018). The primary transcript, of 693 bp, contains two introns of 77 and 84 bp, respectively (Figure 1) (Kotenko et al., 2000; Lockridge et al., 2000). Notably, a region comprising part of the first exon and part of the first intron of the HCMV UL111A is colinear with a previously identified ORF of 79aa, the morphological transforming region (mtrII), involved in rodent cell transformation (Muralidhar et al., 1996). Studies such as these suggest that, under certain conditions, regions of the HCMV genome may have oncogenic properties which needs further research.

An additional shorter HCMV IL10 transcript was also identified in primary human GM-Ps, latently infected with the CMV strains Toledo, AD169 and Towne, and was named latent associated transcript (LAcmvIL-10, also termed the “B” transcript) (Kondo et al., 1996). LAcmvIL-10 was also detected in mononuclear cells from healthy bone marrow and mobilized peripheral blood allograft donors, demonstrating its expression during natural latent infection (Jenkins et al., 2004).

The LAcmvIL-10 transcript results from a single splice event in which only the first intron of the full length cmvIL-10 transcript is removed resulting in the presence of an in-frame stop codon at nucleotide position 160171 in the AD169 strain, 12 amino acids after the end of the second exon (Chee et al., 1990) (Figure 1). Primer-walking RT-PCR assays demonstrated that the LAcmvIL-10 in AD169 starts at a site 38 bp (between nucleotide position 159577 and 159615) upstream of the transcription start site of cmvIL-10 (position 159642) (AD169, accession number X17403), but both LAcmvIL-10 and cmvIL-10 transcripts terminate at the same site (position 160430) and translation of cmvIL-10 and LAcmvIL-10 starts at the same methionine (position 159678). LAcmvIL-10 comprises a truncated protein of 139 aa that shares the first 127 aa residues with the cmvlL10 protein. LAcmvIL-10 has 27% identity and 46% similarity to cIL-10 over a 124 amino acid region (Jenkins et al., 2004).

Interestingly, the LAcmvIL-10 transcript was detected in only 1–12% of latently infected GM-Ps, indicating that latency may still proceed in some cells that fail to express these transcripts or that the limit of their detection was too low (Jenkins et al., 2004). Sometime after the initial identification of LAcmvIL-10 in latently infected cells, the same group showed that LAcmvIL-10 is also expressed during lytic infection of HFF cells at 72 h post-infection. Even though the transcript was shown to be expressed during both lytic and latent infections, the name LAcmvIL-10 is still used (Jenkins et al., 2008a).

Intriguingly, during permissive infection, the LAcmvIL-10 transcript was shown to initiate at the same site as the cmvIL-10 transcript. This likely indicates that the virus utilizes different start sites for LAcmvIL-10 transcription depending on whether the infection is latent or lytic, possibly as a result of differential promoter usage as a result of different cellular and viral factors present in cells at different stages of cell differentiation.

Latterly, five additional UL111A transcripts have been detected in MRC-5 and Bud8 cells productively infected with the AD169 strain by Lin et al. (2008). These transcripts were detected as products of nested PCR by agarose gel electrophoresis and were subsequently cloned and sequenced. In contrast to lytic infection, these transcripts are not detectable to any appreciable extent in infected myelomonocytic THP1 cells in the same work, which are a cell type in which the virus establishes latency (Beisser et al., 2001). In order to facilitate nomenclature, in their work, the cmvIL-10 and LAcmvIL-10 were named transcripts vIL10A and B, respectively, and the additional newly found transcripts were named vIL10C, D, E, F, and G. Donor and acceptor sites were identified in all transcripts with exception of D. Of note, transcript D does not contain the first intron and therefore has the intact 79 aa ORF, which upon translation could produce a putative 79 aa oncogenic protein, previously described.

All identified HCMV vIL10 transcripts share the first exon (nucleotide sequence 159678–159857) at the N terminal but their C terminal region varies in amino acid sequence and length (Figure 1). The report by Lin et al. is the only report of the production of additional spliced transcripts, besides cmvIL-10 and LAcmvIL-10 in HCMV infected cells. It is possible that the absence of their previous detection could be due to the low sensitivity of the techniques used, despite the same primer sets being used as those used in the original identification of cmvIL-10 (Kotenko et al., 2000). Considering the frequency in which the smaller transcripts were found, and the presence of donor and accepting sites in all of them (with exception of transcript D) it would be of considerable interest to analyze these transcripts further during lytic and latent infection.

Interestingly, HCMV UL111A is the only viral cIL-10 homolog that expresses different vIL10 proteins by alternative splicing. Alternatively spliced vIL10 transcripts were not identified in other herpesviruses (Ouyang et al., 2014). This suggests that HCMV may express different transcripts at different phases of infection. This area of research has not been explored and awaits further investigation.

## The HCMV IL10 Proteins, Their Structures and Functions

In cell culture HCMV UL111A is not essential for viral replication (Dunn et al., 2003; Yu et al., 2003), however the gene conservation in HCMV strains, the lack of sequence variability (Cunningham et al., 2010; Sijmons et al., 2014) and the functional analysis of the encoded proteins *in vitro*, described below, indicate that they have critical importance in controlling the host immune system during active infection, persistence and latency.

Consistently, research with Rhesus macaques, the closest CMV to HCMV in which an animal model exists, provided evidence for the role of RhCMV UL111A during infection *in vivo*. The UL111A genes from RhCMV and HCMV are close homologs (Powers and Früh, 2008; Itell et al., 2017) and *in vitro* functional analysis of RhcmvIL10 demonstrated that it has similar properties to HCMV IL10, such as inhibition of PBMC proliferation, inhibition of cytokine production and downregulation of MHC in immune cells (Spencer et al., 2002). RhCMV UL111A, like cmvIL-10 from HCMV, is also not essential for viral growth in cell culture and this is the same for all other viral cIL-10 homologs analyzed so far (Chang and Barry, 2010). However, studies in macaques infected with recombinant viruses showed that the lack of RhCMV UL111A has profound effects in the magnitude of both innate and adaptive host immunity and indicate that RhCMV IL10 is important for dissemination of the virus during primary infection (Chang and Barry, 2010).

## The cmvIL10 Protein

Among the proteins encoded by the HCMV UL111A (Figure 1), the cmvIL-10 (also termed the “A” transcript) protein is the best structurally and functionally characterized. Although expression of the cmvIL-10 transcript and functions of the protein have only been extensively analyzed during the lytic virus life cycle, it has been shown to play a role in the regulation of cIL-10 in cells which support latent infection (Avdic et al., 2016). Indeed, the upregulation of cIL-10 during latent infection in both CD34 and CD14 monocytes has been proposed to play an antiapoptotic role in latently infected cells (Poole and Sinclair, 2015; Poole et al., 2015).

The cmvIL-10 protein is glycosylated, likely at an N linked glycosylation site Asn-151-X-Thr-153. Upon cleavage of the 25 aa leader peptide, the protein is secreted from infected cells (Kotenko et al., 2000; Spencer et al., 2002; Chang et al., 2004) and binds to the IL10 receptor (IL10R) (Kotenko et al., 2000; Spencer et al., 2002), with identical affinity to cIL-10 (Jones et al., 2002), despite their low amino acid sequence identity (27%). In fact, cmvIL-10 is able to compete with cIL-10 for receptor binding (Kotenko et al., 2000). Jones et al. (2002) have reported the crystal structure of cmvIL-10 in complex with the extracellular domain of the IL-10R1, demonstrating that it binds to the receptor in the same intertwined dimer topology as cIL-10. However, while in the cIL-10 dimer, the 2-fold related domains comprise four helices (A–D) from one chain and two helices from the other chain (E and F) (Walter and Nagabhushan, 1995; Zdanov et al., 1995), the cmvIL-10 dimer consists of five alpha helices, comprising helices A, B and D donated from one peptide chain and helices E and F donated from the 2-fold related chain. In addition, the 2-fold related domains of the cmvIL10 dimer adopt a 130° interdomain angle, compared with 90° for cIL-10 (and EbvIL-10), as a result IL-10R1 bound to cmvIL-10 moves 25°, relative to the cIL-10/IL-10R1 complex, toward the putative position of the cell membrane. Despite this peculiar engagement with the receptor, cmvIL-10 uses essentially the same structural epitope as cIL-10, comprised of helix A, the AB loop, and helix F, to contact the IL-10R1 (Jones et al., 2002).

Interestingly, the structural studies of cmvIL-10 and EbvIL10 revealed that even though both mimic the structure of cIL10 their particular engagements with the receptor occur in specific ways, resulting in different receptor affinities and distinct activation of signaling pathways (Liu et al., 1997; Jones et al., 2002).

## Biological Activities of cmvIL-10 in Monocyte Derived Dendritic Cells

In a particularly high affinity interaction with the IL-10 receptor, cmvIL-10 activates the Jak-STAT pathway and, therefore, its immunosuppressive effects can be mediated by phosphorylation of STAT3 (Kotenko et al., 2000; Spencer et al., 2002; Jenkins et al., 2008b). cmvIL-10 has several immunomodulatory properties, particularly in immune cells of the myeloid lineage which are biologically relevant sites of HCMV infection not least because monocytes and their derivatives, macrophages and dendritic cells, are sites of latency and reactivation, respectively (Mendelson et al., 1996; Hahn et al., 1998; Sinclair and Poole, 2014).

DCs play a central role in the orchestration of the immune response and are mainly present in peripheral sites as immature cells, where capture and process antigens are loaded in MHC class I and MHC class II molecules for presentation to effector immune cells. Upon stimuli, such as recognition of PAMPs by pattern-recognition receptors, DCs undergo maturation and migrate to secondary lymphoid organs, becoming the most potent professional APCs and activators of the T cell response (Steinman, 1991; Reis e Sousa, 2001). Substantial work has been carried out to uncover the functions of cmvIL-10 in dendritic cells. cmvIL10 present in the supernatants from HCMV-infected cultures inhibits lipopolysaccharide (LPS)-induced DC maturation, as observed by the reduced levels of maturation markers (including CD83 and HLA-DR as well as CD40, CD80, and CD86), and production of the pro-inflammatory cytokines IL-12, IL-6, and TNF-α. These inhibitory effects are specifically mediated through the IL-10 receptor and are only observed in immature DCs (iDCs) cultured with supernatants from WT virus but not with supernatants from a UL111A deleted virus (Chang et al., 2004). Furthermore, in LPS treated iDCs, recombinant cmvIL-10 blocks expression of IL6, IL-12, TNF alpha (Chang et al., 2004), MHC class I and II, as well as other costimulatory molecules such as CD40, CD80, CD86, B7-H1, B7-DC (Raftery et al., 2004). cmvIL-10 also inhibits cell surface exposure of CD1a, CD1b, and CD1c in DCs (Raftery et al., 2008). Additionally, members of a family of non-classical class I (MHC-I) (which have a role in the presentation of hydrophobic antigens, such as lipids to natural killer T (NKT) cells, a specialized cell type that expresses both NK markers and T-cell receptors on their surface) are also downregulated (Major et al., 2006).

Interestingly, cell adhesion molecules are upregulated (CD44, DC-SIGN) or downregulated (CD11, CD18, and ICAM-1) by cmvIL10 in DCs, suggesting its role in modulation of cell adhesion (Raftery et al., 2004). Furthermore, cmvIL10 upregulates HLA-DM, a non-classical HLA molecule which is part of an unusual extracellular presentation pathway that allows Ag processing and peptide loading outside immature DCs (Santambrogio et al., 1999; Arndt et al., 2000), and IDO, a regulator of T cell proliferation and survival (Raftery et al., 2004). cmvIL10 also plays a role in increasing infectivity of the virus in DCs by increasing expression of DC-SIGN (Raftery et al., 2004), a lectin expressed on the surface of DCs which has been shown to be used by the virus to enter DCs (due to the ability of gB to bind DC-SIGN) (Halary et al., 2002).

Intriguingly, recombinant cmvIL-10 increases apoptosis in activated immature DCs, by blocking the antiapoptotic c-FLIP_L_ and Bcl-x_L_, which are normally upregulated during LPS activation (Raftery et al., 2004). Therefore it is, perhaps, unsurprising that during a lytic infection the virus has evolved ways of counteracting the induction of apoptosis (McCormick et al., 2003). It is also true that during a latent infection in undifferentiated myeloid precursor cells the cellular apoptome is modulated (Poole and Sinclair, 2015), although cIL10 plays an anti-apoptotic role in these cells via the regulation of PEA-15 (Poole et al., 2015). Further effects of viral IL10 during latency are discussed in the myeloid progenitor sections below.

Importantly, cmvIL10 enhances cIL10 expression in DCs (Chang et al., 2004), potentiating the anti-inflammatory effects of cIL-10 (Corinti et al., 2001). Notably, various effects of cmvIL10 on immature DCs, are properties shared with cIL10 and not observed, or observed to a lesser extent, in cells treated with ebvIL10 (Raftery et al., 2004), likely due to the low affinity of ebvIL10 to the cIL10 receptor (Jones et al., 2002).

**Figure 2:**
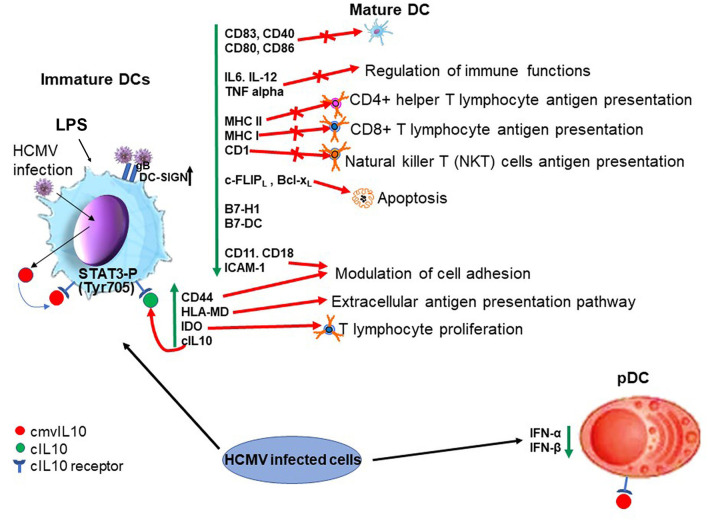
Biological properties of cmvIL10 in dendritic cells (lytic infection). On the left hand side cmvIL10 secreted by infected iDC or by bystander infected cells binds to the cIL10 receptor in iDCs inducing STAT3 phosphorylation and downstream signaling. Green vertical arrows indicate molecules up or downregulated upon signaling and red point to pathways induced or inhibited by cmvIL10. On the right hand side cmvIL10 secreted by infected cells acts though the cIL10R and inhibits production of IFN-α and IFN-β in pDCs.

Together, these studies demonstrate that cmvIL-10 shares a number of known properties of cIL-10 on DCs and these are summarized in Figure 2.

## Biological Effects of cmvIL-10 in Myeloid Progenitors

Significant work has also been carried out to evaluate the effects of cmvIL-10 in myeloid progenitors, cell types that supports latent HCMV infection (Sinclair and Poole, 2014). Recombinant cmvIL-10 inhibits IFN-γ, IL-1α, IL-6, GM-CSF, and TNFalpha (Spencer et al., 2002), by preventing NF-κB signaling in monocytes via inhibition of IKK (Nachtwey and Spencer, 2008), as well as cell surface expression of MHC I and II in stimulated PBMCs, monocytes and GM-Ps (Spencer et al., 2002; Jenkins et al., 2008b). Furthermore, cmvIL-10 suppresses proliferation of PBMCs (Spencer et al., 2002). At least in part, the cmvIL-10 effect in reduction of MHC II protein in monocytes results from downregulation of CIITA, a transcription factor that activates transcription of α, β, and invariant MHC II chains genes leading to the accumulation of MHC-II molecules within cytoplasmic vesicles (Jenkins et al., 2008b). cmvIL-10 also induces upregulation of HLA-G on monocytes (Spencer et al., 2002), a molecule able to confer cell protection from natural killer cell mediated lysis (Rouas-Freiss et al., 1997).

Although it is established that canonical IL-10 signaling in monocytes requires the IL10R and activates STAT3 phosphorylation (Tyr 705) (Jenkins et al., 2008b), JAK1 activity and STAT3 phosphorylation on tyrosine 705 are not required for the inhibition of TNF-α levels by cmvIL-10. Instead, it was shown that TNF-α production requires the PI3K signaling pathway, which culminates with STAT3 phosphorylation on S727 (Spencer, 2007). In fact, as further discussed below, both PI3K and STAT3 are required for cmvIL10 signaling in monocytes (Avdic et al., 2016). The PI3K-mediated activation by cIL10 is well-established and this pathway was thought to be involved in the proliferative effects of the cytokine (Crawley et al., 1996), however recent evidence indicates that PI3K signaling is also required for the immunosuppressive functions of cIL10 (Antoniv and Ivashkiv, 2011).

cmvIL10 is an inducer of cIL10 transcription and protein secretion in CD14+ monocytes, monocyte-derived macrophages (MDMs), and immature monocyte-derived dendritic cells (MDDCs). In monocytes it has been shown that cmvIL10 also induces mRNA expression of tumor progression locus 2 (TPL2) (Avdic et al., 2016), which acts as a regulator of positive and negative feedback loops for cIL-10 production (Saraiva and O'Garra, 2010). It was also reported that cmvIL10 signaling, through the receptor, leads to upregulation of HO-1, a heme-degrading enzyme with immunosuppressive functions (Otterbein et al., 2003), which in turn induces cIL10 in monocytes (Avdic et al., 2016). Since both PI3K and STAT3 are required for cIL10 induction in monocytes (Avdic et al., 2016), the most likely scenario is that these pathways may converge, likely at STAT3 phosphorylation on different residues, leading to activation of STAT3-inducible genes.

**Figure 3:**
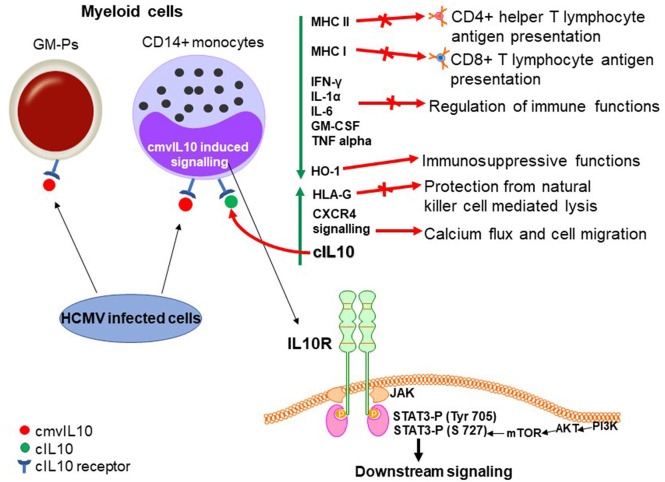
Biological properties of cmvIL10 in myeloid progenitors (during lytic infection). cmvIL10 secreted by bystander infected cells binds to the cIL10 receptor in myeloid progenitors inducing phosphorylation of STAT3 on Tyr 705. cmvIL10 also activates the PI3K pathway leading to phosphorylation of STAT3 on S727. Phosphorylated STAT3 activates downstream signaling. Green vertical arrows indicate molecules up or downregulated upon signaling and red arrows point to pathways induced or inhibited by LAcmvIL10.

Importantly, cmvIL10 appears to have no effect on cIL10 secretion in CD4+, CD8+ T cells or primary human foreskin fibroblasts (HFFs), supporting the fact that myeloid cells are the main targets of cmvIL10 (Avdic et al., 2016). It has also been shown that recombinant cmvIL-10, acting through the cIL10R and STAT3 phosphorylation, can increase CXCR4 signaling mediated by its ligand CXCL12 leading to calcium flux and cell migration in epithelial and monocytic cell lines, in an autocrine and paracrine manner on bystander cells (Tu et al., 2018). These effects are summarized in Figure 3.

## Biological Effects of cmvIL-10 in Non-Myeloid Cells

The effects of cmvIL-10 have also been evaluated in plasmacytoid dendritic cells (pDCs), so called natural interferon-producing cells, a specialized cell population that produces high amounts of type I interferon in response to virus infection cells (Colonna et al., 2004). In pDCs, cmvIL-10, similar to cIL-10, suppresses transcription of IFN-α and IFN-β genes and also affects their steady mRNA levels causing a reduction of IFN-α and IFN-β levels by ~75% (Chang et al., 2009). Also, like cIL10 (Bruchhage et al., 2018), cmvIL-10 is not able to inhibit expression of CD83, CD86, and MHC class II and consequently pDC maturation in response to CpG (Chang et al., 2009).

cmvIL10 has also been shown to stimulate cIL10 and to increase cell survival and proliferation of B cells (Spencer et al., 2008) as well as to decrease metalloproteinase levels in endothelial cells and cytotrophoblasts (a cell type present in the uterine-placental interface) leading to impaired invasion and migration capacities and consequently dysregulation of the cell-cell and/or cell-matrix interactions between these cells (Yamamoto-Tabata et al., 2004).

In some cancer cell types cmvIL10 has been shown to stimulate proliferation and migration (Bishop et al., 2015) and to promote invasiveness (Valle Oseguera and Spencer, 2014, 2017).

## The LAcmvIL10 Protein and Its Biological Effects in Myeloid Progenitors

The LAcmvIL10 protein is produced with β kinetics in lytic infection (Jenkins et al., 2008a) and contrary to cmvIL10 is not glycosylated due to absence of the Asn^151^-Gly^152^-Thr^153^- glycosylation site present in the C terminus of cmvIL10 (Jenkins et al., 2008a; Lin et al., 2008).

LAcmvIL10 has been less extensively studied than cmvIL10 and most studies have focused on the properties of the protein in cell models of latency *in vitro*, since it was the originally detected in latent HCMV infected cells (Jenkins et al., 2004). Similar to cmvIL10, recombinant LAcmvIL10 causes a decrease in total MHC-II protein and transcription of components of the MHC class II biosynthesis pathway in GM-Ps and monocytes (Jenkins et al., 2008b).

In the context of viral infection, LAcmvIL10 inhibits IFN-γ production in CD4^+^ T cells and their capacity to proliferate, as well as to recognize CD34+ cells latently infected with HCMV (Cheung et al., 2009). However, MHC class II downregulation by LAcmvIL10 is not blocked by neutralizing antibodies to cIL10R and does not trigger STAT3 phosphorylation of Tyrosine 705 (Jenkins et al., 2008b). Additionally, LAcmvIL10 does not inhibit the expression of costimulatory molecules CD40, CD80, and CD86 and the maturation marker CD83 on DCs, nor does it inhibit proinflammatory cytokine expression (IL-1, IL-6, and tumor necrosis factor alpha) (Jenkins et al., 2008b). Also, in comparison to cmvIL10, it is not able to affect expression of Fcγ receptors or increase receptor mediated phagocytosis in monocytes (Jaworowski et al., 2009). Additionally, in contrast to cmvIL10, LAcmvIL10 does not stimulate B cell proliferation, Stat3 activation or cIL-10 production in B cells a non-myeloid cell type (Spencer et al., 2008).

It is already established that the restricted signaling abilities of LAcmvIL10 are likely due to the lack of the C-terminal helices E and F (Jenkins et al., 2008b), which are present in cmvIL10 and cIL10 and are required for binding to the IL10R (Walter and Nagabhushan, 1995; Zdanov et al., 1995; Jones et al., 2002). Furthermore it is suggested that LAcmvIL10 engages the receptor in a different manner, utilizes a different receptor or uses a receptor-independent mechanism for downregulation of MHC class II (Jenkins et al., 2008b). Therefore, further studies are required to understand if LAcmvIL10 is still able to engage the receptor, how it downregulates MHC class II in latent cells and whether it has additional properties in both lytic and latent HCMV infection.

Analyses of recombinant HCMV vIL10 proteins (LAcmvIL10 and cmvIL10) as well as comparative studies using supernatant from HFFs infected with the AD169 and Merlin with and without UL111A, showed that the viral IL10 proteins cause polarization of CD14+ monocytes toward an M2c alternatively activated phenotype, as determined by increase of cell surface expression of CD14 and CD163 and decreased of MHC class II (Avdic et al., 2013), markers characteristic of M2c activated monocytes (Gordon, 2003; Mantovani et al., 2004; Zizzo et al., 2012). Furthermore, in polarized monocytes, vIL10 proteins cause a reduction of TNF-α and IL-1β, by upregulation of HO-1 (Avdic et al., 2013), although it remains to be established which of the viral IL10 proteins predominantly mediate these effects.

**Figure 4:**
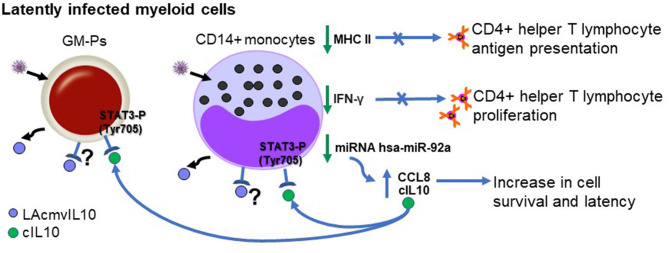
Biological properties of LAcmvIL10 in myeloid progenitors (during latent infection). Infected myeloid cells produce LAcmvIL10. Green vertical arrows indicate molecules up or downregulated upon LAcmvIL10 by an undefined mechanism. Blue arrows point to pathways induced or inhibited by cIL10. The question mark indicates the possibility that LAcmvIL10 can act through the cIL10R.

In addition, studies using the clinical isolate stain of HCMV Merlin, showed that the lack of UL111A led to a decrease in the establishment of latent infection of CD14^+^ monocytes, as well as in latently infected CD34^+^cells (Poole et al., 2014), suggesting that LAcmvIL10 may play a role in the establishment and/or maintenance of latency. LAcmvIL10 has also been shown to cause downregulation of the cellular miRNA hsa-miR-92a leading to increase of secreted cIL10 and CCL8, which are direct targets of this miRNA (Poole et al., 2014). CCL8 acts to subvert the immune response and, thus, is likely to be important for the establishment of latency *in vivo* (Mason et al., 2012). The effects of LAcmvIL10 are summarized in Figure 4.

The LAcmvIL-10-induced increase in cIL10 appears to have an important role in survival of CD14+ monocytes and CD34+ precursor cells, and consequently in the establishment of viral latency in the cells, as shown by a decrease in latent carriage of genome CD14+ monocytes and CD34+ precursor cells in the absence of cIL10, suggesting a role for IL10 in the maintenance of HCMV latency (Poole et al., 2011). Enhanced cell survival in myeloid progenitors during HCMV latency is also a property of cIL10 (Poole and Sinclair, 2015; Poole et al., 2015).

## Proteins Encoded by the Additional HCMV IL10 Transcripts

The proteins encoded by the UL111A transcripts C, D, E, F, and G share the first 60 N terminal aa, with cmvIL10 (A) and LAcmvIl10 (B), containing a signal peptide of 19 aa, but with C terminal region varying in amino acid sequence and length. The sizes of the transcripts and aa length of the proteins are shown in Figure 1. These proteins were first identified in lytically infected MRC-5 cells by western blotting (Lin et al., 2008). Isoforms vIL-10A, E, and F were found to be glycosylated, but not B, C, and D, consistent with the presence or absence of the N-linked glycosylation site, Asn-151-X-Thr-153, in their sequences. As in the case of LAcmvIL10 (B transcript) none of the isoforms identified were able to induce STAT3 phosphorylation in THP1 cells (Lin et al., 2008).

Further work is necessary to verify presence of these additional isoforms in other cell types and if they have any immunosuppressive or biological properties that influence viral carriage and propagation.

## Concluding Remarks

The HCMV vIL10 proteins have a variety of immunosuppressive properties in different cell types. The two most studied isoforms, cmvIL10 and LAcmvIL10 (transcripts A and B) appear to be differentially expressed in lytic and latent infected cells. LAcmvIL10 has restricted functions compared to cmvIL10, and one of its properties is downregulation of MHC II, aiding immune evasion of the virus by inhibiting presentation of viral antigens expressed during latency (Table 1).

**Table 1:** Biological properties of cIL-10 and HCMV vIL-10.

	**Gene expression (kinetics)**	**STAT3 activation**	**Cytokine inhibition**	**MHC inhibition**
cmvIL-10 (A)	Lytic infection (γ)	Yes	Yes	Yes
LAcmvIL-10 (B)	Lytic infection (β) Latent infection	No	No	Yes
C	?	No	?	?
D	?	No	?	?
E	?	No	?	?
F	?	No	?	?
G	?	No	?	?
cIL-10	NA^##^	Yes	Yes	Yes

*?, not known*;

## *NA, not applicable*.

The production of different vIL10 isoforms by HCMV, with different structures and specific biological properties indicate that the virus evolved to use these cIL10 homologs in different phases of infection.

Since their original identification, a great deal of work which we have detailed in this review, has been carried out to analyze the expression of viral IL-10 transcripts, the proteins encoded by these RNAs and the biological functions of these proteins. As much of this has depended on the analysis of purified recombinant proteins, further studies, ideally using clinical isolate recombinant viruses, are necessary to fully understand the biological properties of the all the viral IL10 proteins in different cell types and at stages of the virus life cycle.

## Author Contributions

EP, JS, and MS contributed with discussion and writing. TN, MO, and MS contributed with the figures and table of the final manuscript.

## Conflict of Interest

The authors declare that the research was conducted in the absence of any commercial or financial relationships that could be construed as a potential conflict of interest.
